# A Diagnosis Model of Typhoon‐Related Post‐Traumatic Stress Disorder Based on Fixel‐Based Analysis in Machine Learning

**DOI:** 10.1002/brb3.71354

**Published:** 2026-04-06

**Authors:** Yiying Zhang, Huijuan Chen, Rongfeng Qi, Jun Ke, Qiang Xu, Yuan Zhong, Li Zhang, Guangming Lu, Feng Chen

**Affiliations:** ^1^ Department of Radiology, Hainan General Hospital Hainan Affiliated Hospital of Hainan Medical University, Hainan Medical University Haikou Hainan China; ^2^ Department of Medical Imaging, Jinling Hospital Medical School of Nanjing University Nanjing Jiangsu China; ^3^ Department of Radiology The First Affiliated Hospital of Soochow University Suzhou Jiangsu China; ^4^ School of Psychology Nanjing Normal University Nanjing Jiangsu China; ^5^ Department of Psychiatry, Second Xiangya Hospital Central South University; China National Clinical Research Center on Mental Disorders (Xiangya), China National Technology Institute On Mental Disorders, Hunan Key Laboratory of Psychiatry and Mental Health Changsha China

**Keywords:** fixel‐based analysis, imaging of diffusion tensor, machine learning, microstructure of white matter, post‐traumatic stress disorder

## Abstract

**Background:**

Post‐traumatic stress disorder (PTSD) is the most common mental disorder following traumatic experiences. Environmental disasters such as super typhoons can severely disrupt daily life and may trigger PTSD in exposed individuals. White matter alterations have been observed in patients with PTSD. Fixel‐based analysis (FBA), a recently developed diffusion MRI technique, allows detailed assessment of white matter microstructure. This study aimed to evaluate the potential of FBA as an imaging biomarker in typhoon survivors, reducing the subjective bias associated with clinical symptom scales.

**Methods:**

Whole‐brain diffusion MRI data from the PTSD group (*n* = 27), trauma‐exposed controls (TEC, *n =* 33), and healthy controls (HC, *n =* 30) were analyzed to identify white matter fiber tracts showing abnormalities in FBA metrics, including fiber density (FD), fiber cross‐section (FC), and fiber density–cross section (FDC). The study then examined whether these FBA‐derived features, when combined with machine learning, could improve the identification of potential PTSD biomarkers.

**Results:**

Compared with the HC group, patients with PTSD showed increased fiber density (FD) in the right frontopontine tract and right middle longitudinal fascicle, as well as higher fiber density–cross section (FDC) values in the bilateral frontopontine tract and left thalamo‐premotor tract (Bonferroni correction, p < 0.05/18 = 0.003). To differentiate PTSD from TEC, binary and multiclass machine learning models with five‐fold cross‐validation were developed. The binary model (PTSD vs. TEC) achieved high performance (accuracy = 0.89, sensitivity = 0.97, specificity = 0.71, precision = 0.87, AUC = 0.95), whereas the multiclass model (PTSD vs. TEC vs. HC) demonstrated excellent results (macro‐averaged precision = 0.99, recall = 0.99, F1‐score = 0.99). The top 20 contributing features of the optimal model were analyzed using Shapley additive explanation (SHAP) values to illustrate model interpretability.

**Conclusion:**

Most typhoon‐exposed individuals with PTSD may exhibit structural alterations in brain white matter. By combining fixel‐based analysis (FBA) with machine learning, this study identified diffusion markers within specific white matter tracts and demonstrated their potential diagnostic value for distinguishing PTSD from trauma‐exposed controls. These findings enhance our understanding of microstructural white matter changes and their spatial distribution in PTSD and also suggest potential imaging biomarkers for its diagnosis.

## Graphical abstract

Fixel‐based analysis revealed discrete patterns of white‐matter disruption in typhoon‐related PTSD, particularly affecting the frontopontine, middle longitudinal, and thalamo‐premotor pathways. Integrating these tract‐specific signatures into machine‐learning models allowed robust discrimination between PTSD and trauma‐exposed controls, suggesting their potential utility as reproducible and biologically grounded imaging biomarkers.

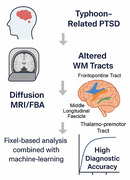

## 1 Introduction

Post‐traumatic stress disorder (PTSD) is a psychological condition that may develop in individuals exposed to extreme or prolonged stress (Edition 2013). Traumatic events such as military conflicts, terrorist attacks, major traffic accidents, and natural disasters, including earthquakes and typhoons, can precipitate PTSD. Evidence from naturalistic disaster cohorts, such as avalanche survivors, further demonstrates substantial variability in PTSD prevalence and associated risk factors following large‐scale traumatic exposure (Kurhan et al. 2023). The disorder is characterized by four main symptom clusters: re‐experiencing, avoidance, negative alterations in cognition and mood, and hyperarousal. Pathophysiological models of PTSD have evolved from focusing on frontal–limbic circuitry to encompassing large‐scale neural networks in the brain (Ross and Cisler 2020), with white matter (WM) tracts serving as essential pathways linking gray matter regions (Ju et al. 2020). Compared with the relatively well‐studied gray matter atrophy observed in PTSD, current understanding of WM pathology remains limited. WM alterations may represent potential biomarkers of the disorder. Diffusion tensor imaging (DTI) has been widely applied to characterize WM microstructure and to explore neural mechanisms underlying PTSD. However, in regions where fiber tracts intersect or merge, conventional DTI indices such as fractional anisotropy (FA) show limited specificity in detecting microstructural alterations (Jones and Cercignani 2010; Lehman et al. 2011; Wheeler‐Kingshott and Cercignani 2009).

DTI, which is sensitive to the diffusion of water molecules, has been widely applied to investigate potential WM abnormalities in PTSD (Daniels et al. 2013; Hans 2011). The WM fiber tracts implicated in memory, attention, emotional regulation, and language processing are often affected. Biological studies have suggested that different PTSD subtypes may involve distinct pathophysiological mechanisms and a range of potential biomarkers (Huckins et al. 2020). Early‐life trauma has been shown to exert long‐term effects on neurobiological vulnerability, potentially shaping stress sensitivity and clinical outcomes following later trauma exposure (Yilgör and Kurhan 2024). Similarly, large‐scale disasters such as the COVID‐19 pandemic have been associated with widespread psychological distress and transdiagnostic vulnerability patterns, highlighting substantial heterogeneity in stress responses across exposed populations (Cim et al. 2022). However, findings on the association between PTSD and WM abnormalities have not been entirely consistent. DTI studies have reported that brain regions critical for fear learning and memory are interconnected through the cingulum bundle, uncinate fasciculus, and fornix/stria terminalis, implying that mood disturbances in PTSD may be directly linked to microstructural alterations within these tracts (Fani et al. 2016; Fani et al. 2019; Harnett et al. 2020). Furthermore, both the direction and location of WM abnormalities vary across studies: decreased and increased FA values have been observed in the posterior and dorsal cingulate gyrus, respectively (Siehl et al. 2018). Previous studies also indicated that FA values within the uncinate fasciculus correlate with PTSD symptom severity (Costanzo et al. 2016; Harnett et al. 2020; O'Doherty et al. 2018). Thus, the microstructural integrity of the uncinate fasciculus may mediate the impact of traumatic stress on amygdala–prefrontal cortical interactions.

However, in regions where multiple fiber tracts intersect, voxel‐averaged diffusion measures such as FA and MD often fail to accurately characterize the underlying microstructure, as they cannot distinguish distinct fiber populations within a voxel. Fixel‐based analysis (FBA) is an advanced diffusion MRI technique that overcomes the limitations of traditional voxel‐based analyses (VBA). By modeling individual fiber populations, or ‘fixels,’ within each voxel, FBA enables precise quantification of fiber‐specific microstructural alterations, addressing challenges posed by complex fiber geometries in regions of crossing fibers. This approach provides more accurate and physiologically relevant insights into white matter microstructure compared to conventional methods (Raffelt et al. 2017). This method allows for bundle‐specific comparisons at the subvoxel level and enables the quantification of multiple fiber orientations within a single voxel. FBA overcomes several drawbacks of traditional DTI metrics by providing more specific estimates of white matter microstructural alterations within individual fiber bundles. FBA overcomes the inherent direction ambiguity of voxel‐averaged approaches. Built on fiber orientation distributions (FODs), FDA enables biologically interpretable decomposition of white matter alterations into fiber density (FD), reflecting microstructural axonal properties, fiber cross‐section (FC), indexing macroscopic bundle morphology, and their combined effect (FDC). Reduced FD or FC values may indicate axonal degeneration or reduced tract integrity (Raffelt et al. 2017). This technique has been successfully applied to several neurological and psychiatric disorders, including Parkinson's disease (Rau et al. 2019), schizophrenia (Verhelst et al. 2019), traumatic brain injury (Verhelst et al. 2019; Wallace et al. 2020), and multiple sclerosis (Carandini et al. 2021; Storelli et al. 2021). However, applications of FBA in PTSD remain very limited.

Beyond neural circuit‐level models, accumulating evidence indicates that trauma‐related psychopathology is accompanied by systemic biological dysregulation extending beyond the central nervous system (Kurhan and Alp 2021b). Studies across stress‐related and psychiatric conditions have consistently reported altered oxidative stress balance, oxidative DNA damage, and impaired redox homeostasis following traumatic exposure. In particular, investigations of thiol/disulfide homeostasis and oxidative DNA damage in PTSD and other trauma‐related disorders suggest that chronic stress induces widespread biological vulnerability that may influence neural integrity. These systemic processes provide a plausible biological context for white‐matter microstructural alterations observed using diffusion MRI, suggesting that imaging markers such as fiber density and fiber cross‐section may reflect downstream or parallel manifestations of broader stress‐related biological cascades rather than isolated neuroanatomical abnormalities.

Consequently, in the present study we employed FBA to examine both micro‐ and macrostructural white matter changes in individuals with typhoon‐related PTSD. Using diffusion‐derived FBA metrics, we compared white matter integrity among patients with PTSD, trauma‐exposed controls (TEC), and healthy controls (HC). To further explore the discriminative potential of these diffusion markers, recursive feature elimination with cross‐validation (RFECV) was integrated with machine learning for group classification. Previous studies have suggested that white matter abnormalities may serve as potential early biomarkers of PTSD. Therefore, this study aimed to identify PTSD‐related white matter alterations and evaluate their diagnostic efficiency through a combination of advanced diffusion imaging.

## 2 Materials and Methods

### 2.1 The Participants and Their Clinical Evaluations

The Medical Ethics Committee of Hainan Affiliated Hospital of Hainan Medical University (Hainan General Hospital) and the Ethics Committee of the Second Xiangya Hospital of Central South University approved this study (Approval No. 20140306), and written informed consent was provided by all participants. All procedures involving human participants were conducted in accordance with the ethical standards of the institutional research committee and the Declaration of Helsinki. The PTSD and TEC groups had the following inclusion criteria: (1) being aged between 18 and 65 years; (2) having been exposed to a super typhoon traumatic event 3 to 6 months before the study; (3) meeting the criteria of the Diagnostic and Statistical Manual of Mental Disorders IV (DSM—IV) for PTSD. The general exclusion criteria for all participants were as follows: (1) a history of a clearly diagnosed neurological, cardiovascular, hepatic, renal, or endocrine disorder, or a history of using psychotropic drugs; (2) a history of alcohol or opioid abuse; (3) clear brain injury, or a history of being unconscious for over 5 min. (4) left‐handedness; (5) contraindications to MRI scanning; (6) excessive head movement during the MRI scan (head movement translation >1.5 mm or rotation angle >1.5°).

On July 18, 2014, south China's coast was hit by the category 5 super typhoon Rammasun, which brought substantial damage to Wenchang in Hainan province, among which Wenchang Luo Dou Farm was included. More than a thousand people were trapped by the typhoon‐induced storm surge and were on the verge of drowning, and at least fourteen people lost their lives. People in the area also endured great economic losses and psychological harm. In November 2014, 102 volunteers who had been through the major natural disaster in Luodu Town, Wengtian Town, and Fengpo Town—the most severely stricken areas in Wenchang—were recruited for this study. The PTSD Checklist Civilian Version (PCL) (Weathers et al. 1994), a 17‐item self‐report questionnaire that measures the intensity of PTSD symptoms on a 5‐point scale ranging from 1 (none) to 5 (extreme) as per the DSM‐IV, was used to screen all the volunteers. For those with scores over 35, DSM‐IV‐TR Axis I Disorders (Structured Clinical Interview for DSM‐IV‐TR Axis I Disorders, Research Version: Patient Edition, SCID‐I/P) (Michael et al. 2002; Weng et al. 2018) was utilized to evaluate whether they met the diagnostic criteria for PTSD and other psychiatric disorders. The diagnosis of PTSD was made in accordance with the existing DSM‐IV standards. Symptoms were evaluated with the Clinician‐Administered PTSD Scale (CAPS) (Weathers et al. 2001). The CAPS is a structured interview that employs a behavioral anchor scale (ranging from 0 to 4) for the assessment of the frequency and intensity of every PTSD symptom. The 17 central PTSD symptoms as listed in the DSM‐IV are evaluated by this scale, and it offers details about how long the symptoms last, when they start, and what impact they have on functions. In addition, 32 healthy people not meeting DSM‐IV diagnostic criteria were recruited via advertisements in the typhoon‐stricken cities. The symptoms of anxiety and depression of all participants were assessed by the Self‐Rating Anxiety Scale (SAS) (Zung 1965) and Self‐Rating Depression Scale (SDS) (Zung 1971). Eventually, 9 individuals with PTSD were removed. Among them, there were 3 without valid imaging data, 2 with metal dental prostheses, 1 having a cerebral infarction, 1 who was pregnant, and 2 with excessive head movement (1 male and 1 female). Moreover, one female volunteer and two male volunteers were removed because of excessive head movement and having a cerebral infarction, respectively. In the end, 90 participants were included in the study, with 27 having PTSD, 33 being TECs, and 30 being HCs.

### 2.2 MRI Scanning Protocols

Structural images, three‐dimensional T1‐weighted images, and diffusion tensor imaging (DTI) data were acquired using a 3.0 T MR scanner (Skyra, Siemens Healthineers, Erlangen, Germany) equipped with a 32‐channel head coil. Foam padding was used to minimize head motion during scanning.

High‐resolution sagittal three‐dimensional T1‐weighted structural images were acquired using a magnetization‐prepared rapid acquisition gradient‐echo (MPRAGE) sequence. The imaging parameters were as follows: repetition time (TR) = 2300 ms, echo time (TE) = 1.97 ms, inversion time (TI) = 900 ms, flip angle = 9°, field of view (FOV) = 256 × 256 mm^2^, matrix = 256 × 256, slice thickness = 1 mm, number of slices = 176, and voxel size = 1 × 1 × 1 mm^3^. These images were used for subsequent co‐registration and normalization. Each scan lasted 353 s. DTI data were acquired using a single‐shot spin‐echo echo‐planar imaging (SE‐EPI) sequence with 30 non‐collinear directions (b = 1000 s/mm^2^) and a reference image without diffusion weighting (b = 0). Data acquisition parameters were as follows: TR/TE = 9000/90 ms, matrix = 128 × 128, FOV = 256 × 256 mm^2^, slice thickness = 2 mm, number of slices = 75, number of excitations = 2, and voxel size = 2 × 2 × 2 mm.

### 2.3 Preprocessing of Magnetic Resonance Images and Fixel Analysis

Diffusion MRI data were preprocessed using MRtrix3 software (https://www.mrtrix.org/) following the publicly available fixel‐based analysis (FBA) pipeline. Standard preprocessing steps included image denoising (Veraart et al. 2016), Gibbs‐ringing artifact removal (Kellner et al. 2016), correction for motion and eddy current‐induced distortions (Andersson and Sotiropoulos 2016), bias field correction (Tustison et al. 2010), skull stripping, and upsampling to 1‐mm isotropic resolution. Fiber orientation distributions (FODs) for white matter, gray matter, and cerebrospinal fluid were estimated using the single‐shell three‐tissue response function estimation method (Dhollander and Connelly 2016), followed by multi‐tissue constrained spherical deconvolution (MSMT‐CSD). To ensure consistency across participants, a group‐averaged response function was applied. The resulting FOD images were then globally intensity‐normalized to allow direct comparison of FOD amplitudes among subjects. Spatial correspondence across individuals was established using FOD‐based nonlinear registration, aligning each individual FOD image to a study‐specific population FOD template (Raffelt et al. 2011). Whole‐brain probabilistic tractography was subsequently performed in the template space to generate a population tractogram (Tournier et al. 2019), which served as the anatomical basis for fixel‐wise analysis. Fixel‐wise metrics were derived, including fiber density (FD), fiber cross‐section (FC), and the combined measure of fiber density and cross‐section (FDC). Because FC values did not follow a normal distribution, logarithmic transformation (logFC) was applied prior to statistical analysis. For region‐based analyses, seventy‐two major white matter tracts were automatically segmented in the template space using TractSeg (https://github.com/MIC‐DKFZ/TractSeg) (Wasserthal et al. 2018). Mean FD, logFC, and FDC values were extracted for each region of interest. All data processing and visualization were conducted on the NeuroScholar Cloud Platform (http://www.humanbrain.cn).

### 2.4 Statistical Analysis

#### 2.4.1 Population and Clinical Features

Categorical variables were analyzed using the chi‐square test. For continuous variables other than the PCL score, one‐way analysis of variance (ANOVA) was performed, and independent t‐tests were used to examine differences between the PTSD and TEC groups. All statistical analyses were conducted using SPSS software (version 21.0; IBM Corp., Armonk, NY, USA).

#### 2.4.2 Analysis Based on the Global

MRtrix3 (version 3.0.2; www.mrtrix.org) (Tournier et al. 2019) was used for image‐based statistical analysis. Participants in the PTSD, TEC, and HC groups were compared across all white matter fixels. To examine group differences in fixel‐wise metrics, including fiber density (FD), log‐transformed fiber cross‐section (logFC), and fiber density and cross‐section (FDC), a general linear model (GLM) was applied. Age, sex, and years of education were included as covariates. Statistical significance was determined using connectivity‐based fixel enhancement (CFE) with 5,000 non‐parametric permutations (D. A. Raffelt et al. 2015). To account for the large number of comparisons made during the Fixel‐based analysis, we applied family‐wise error (FWE) correction, ensuring robust control for Type I error. In addition, we employed 5000 non‐parametric permutations to strengthen the statistical validity of our findings, and all results were considered significant at *p* < 0.05 following this correction.

#### 2.4.3 ROI Analysis

TractSeg (https://github.com/MIC‐DKFZ/TractSeg) (Wasserthal et al. 2018) was used to perform region‐of‐interest (ROI) analyses based on 72 predefined white matter tracts. Fixel‐based metrics, including fiber density (FD), log‐transformed fiber cross‐section (logFC), and fiber density and cross‐section (FDC), were extracted for each tract. Analysis of variance (ANOVA) was used to compare mean values of these metrics among the PTSD, TEC, and HC groups. Post hoc t‐tests were conducted to identify pairwise group differences. Bonferroni‐corrected p‐values were applied to adjust for multiple comparisons.

#### 2.4.4 Classification in Machine‐Learning

FBA‐derived metrics were used to classify participants among the HC, TEC, and PTSD groups. Three fixel‐based indices were extracted from each of the 72 white matter tracts, resulting in 216 features that served as inputs for the classification analysis. To reduce dimensionality and minimize overfitting, recursive feature elimination with cross‐validation (RFECV) was applied using a Random Forest classifier (RandomForestClassifier, scikit‐learn) as the embedded estimator. The model was trained on the full feature set, evaluated feature importance, and iteratively removed the least informative features. This process was repeated within the cross‐validation loop until an optimal subset of features was obtained. The selected features were then used to train the final Random Forest classifier for three‐class classification.

To evaluate model performance and minimize overfitting, five‐fold cross‐validation was applied. A series of models were generated across the five folds. The area under the receiver operating characteristic curve (AUC) was calculated to quantify classification performance. For multiclass classification, both macro‐ and micro‐averaged AUC values were computed. To reduce sampling bias, the cross‐validation procedure was repeated five times for both binary and multiclass models, and the mean ROC curve and average AUC were obtained. Model performance was further evaluated in terms of accuracy, sensitivity, specificity, precision, and recall. Feature importance within the optimal classification model was assessed using Shapley additive explanation (SHAP) values, which estimate the contribution of each feature by averaging its marginal effect across all possible feature combinations. For the multiclass model, mean absolute SHAP values were calculated by averaging the absolute SHAP values across classes and comparing them to the overall model mean.

## 3 Results

### 3.1 Characteristics Related to Demography and Clinical Aspects

Table 1 summarizes the demographic and clinical characteristics of the participants. No significant differences were found in age (*p =* 0.729) or sex distribution (*p* = 0.912) among the TEC, HC, and PTSD groups. However, education differed significantly across groups (*p* < 0.001). Post hoc analysis revealed that the HC group had more years of education than both the PTSD (*p* < 0.001) and TEC (*p* < 0.001) groups, whereas no difference was observed between the PTSD and TEC groups (*p* = 0.518). The mean total CAPS score in the PTSD group was 78.2 ± 19.3, and the PCL score was significantly higher in the PTSD group compared with the TEC group (*p* < 0.001). No participant in the HC group was diagnosed with depression (SAS, *p* < 0.001; SDS, *p* < 0.001). Post hoc comparisons showed that SAS (*p* = 0.025) and SDS (*p* = 0.003) scores in the TEC group were significantly higher than those in the HC group but significantly lower than those in the PTSD group (*p* < 0.001).

**TABLE 1:** Data regarding the demography and clinical situation of both traumatized individuals and healthy controls.

		PTSD (*n* = 27)	TEC (*n* = 33)	HC (*n* = 30)	*p*‐value
Age (years, Mean ± SD)		48.4 ± 10.3	48.5 ± 7.5	49.9 ± 6.1	0.729
Sex (No., %)	males	7 (25.93)	7 (21.21)	7 (23.33)	0.912
	females	20 (74.07)	26 (78.78)	23 (76.67)	
Education (years, Mean ± SD)		6.4 ± 3.4	7.0 ± 3.4	9.7 ± 3.3	<0.001*
SDS score		69.6 ± 13.2	41.3 ± 9.1	33.5 ± 7.2	<0.001*
SAS score		65.8 ± 13.3	41.3 ± 8.1	36.0 ± 5.5	<0.001*
PCL score		53.7 ± 8.5	28.9 ± 5.4		<0.001*
CAPS total score		78.2 ± 19.3			

Abbreviations: CAPS, Clinician‐Administered PTSD Scale; HC, Health control; PCL, PTSD Checklist‐Civilian Version; PTSD, Post‐traumatic stress disorder; SAS, Self‐Rating Anxiety Scale; SDS, Self‐Rating Depression Scale; TEC, Trauma‐exposed control.

*p < 0.Significant differences were indicated by 05.

### 3.2 Analysis Based on Whole‐Brain Fixel

In the FBA, age, sex, and years of education were included as covariates. A one‐way analysis of variance (ANOVA) was performed on fiber cross‐section (FC), fiber density (FD), and fiber density and cross‐section (FDC) across the three groups. No significant group differences were observed after family‐wise error (FWE) correction (*p* < 0.05).

### 3.3 ROI Analysis

**FIGURE 1:**
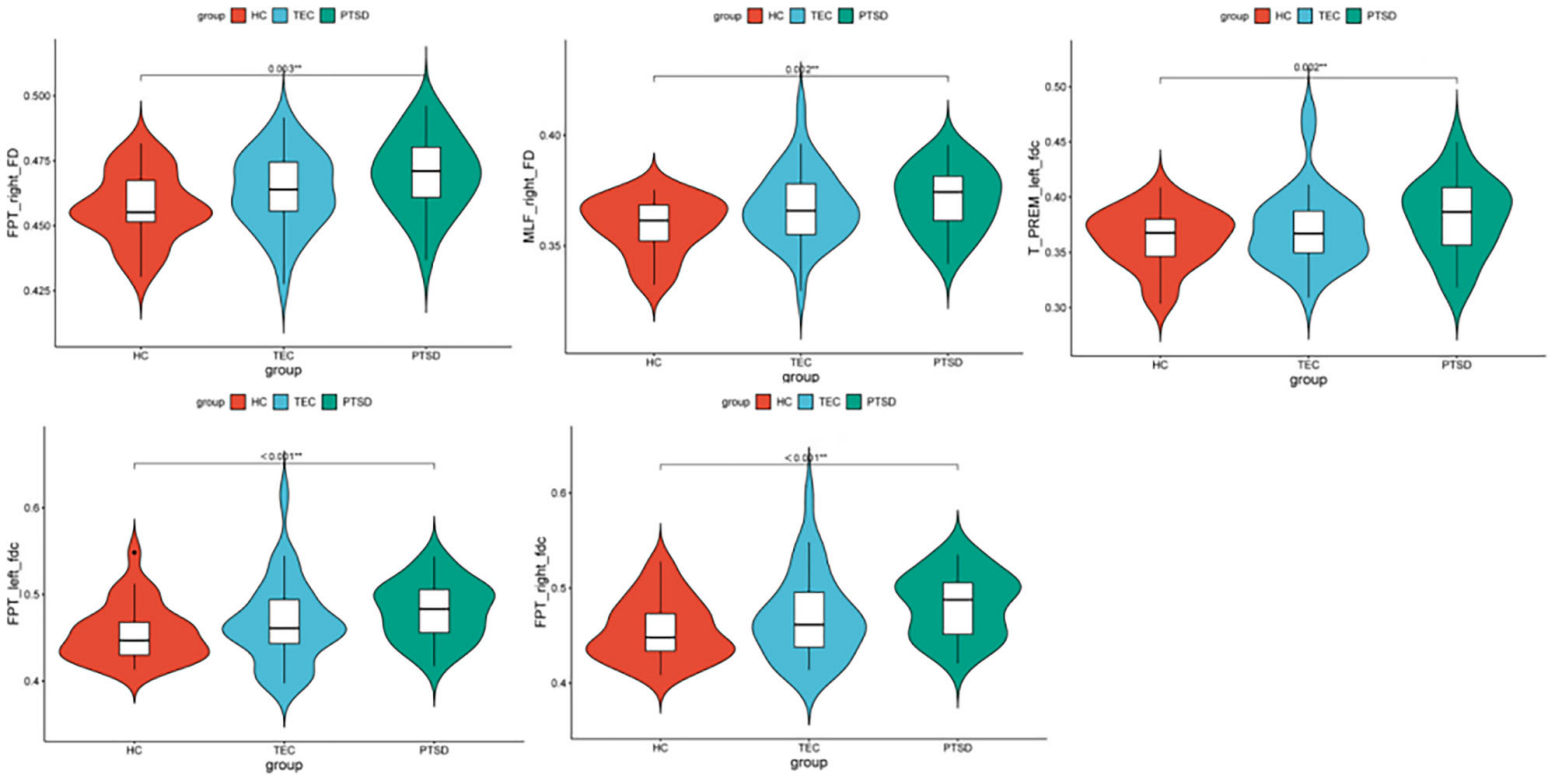
There are remarkable differences among groups in terms of FD and FDC as shown in the tract profiles. Covariates included age, sex, and years of education. In the PTSD group as opposed to the HC group, a statistically significant increase in FD was observed in the right FPT and right MLF. Moreover, compared to the HC group, PTSD patients had higher values in the bilateral FPT and left T_PREM in terms of FDC.

**FIGURE 2:**
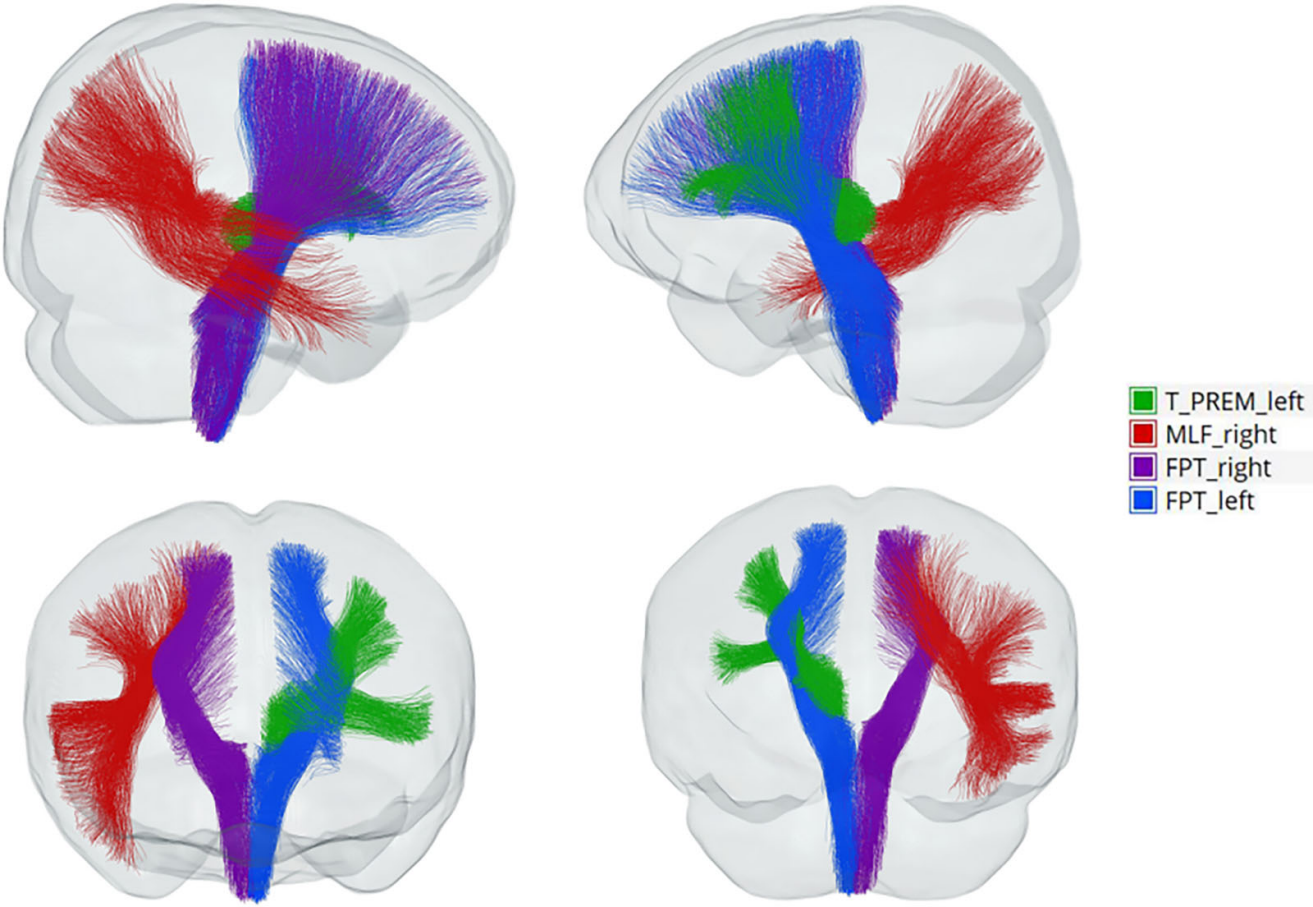
White matter fiber tracts showing group differences. There are four perspectives regarding the differentiated white matter fiber bundles. The frontopontine tract is abbreviated as FPT. The middle longitudinal fascicle is abbreviated as MLF. The thalamo‐premotor is abbreviated as T_PREM.

The ANOVA revealed 18 fiber bundles showing significant group differences in FBA metrics. These tracts and their corresponding measures are summarized in Table 2. These included the right anterior thalamic radiation (ATR) in terms of fractional anisotropy (FA), bilateral frontopontine tracts (FPT), the right middle longitudinal fasciculus (MLF), bilateral parieto‐occipital pontine tracts (POPT), the left superior cerebellar peduncle (SCP), the right prefrontal–striatal tract (PREF_ST), the right thalamo‐parietal tract (T_PAR), the left thalamo‐postcentral tract (T_POSTC), and the left thalamo‐precentral tract (T_PREC). Differences were also found in both thalamo‐precentral regions (T_PREF), the left thalamo‐premotor tract (T_PREM), and the fiber density and cross‐section (FDC) of the bilateral frontoparietal tracts (FPT). The left corticospinal tract (CST) also showed alterations (Table 2). Post hoc comparisons (Bonferroni correction, *p* < 0.05/18 = 0.0028) revealed that, compared with the HC group, patients with PTSD had significantly higher fiber density (FD) values in the right frontopontine tract (FPT) and right middle longitudinal fasciculus (MLF). Other tracts also showed higher FD or FDC values in the PTSD group compared with controls, as summarized in Table 3 and illustrated in Figure 1. The fiber bundles showing significant white matter alterations are displayed in Figure 2a. No significant differences in log‐transformed fiber cross‐section (logFC) were found between the PTSD and HC groups. Likewise, no statistically significant differences in FD, logFC, or FDC were observed between the PTSD and TEC groups or between the TEC and HC groups (Table 3, Figure 1).

**TABLE 2:** The values of fiber tracts in FBA are significantly different.

Fiber tracts	*p*‐value
ATR_right_FD	0.039
FPT_left_FD	0.008
FPT_right_FD	0.006
MLF_right_FD	0.011
POPT_left_FD	0.024
POPT_right_FD	0.017
SCP_left_FD	0.040
ST_PREF_right_FD	0.030
T_PAR_right_FD	0.019
T_POSTC_left_FD	0.021
T_PREC_left_FD	0.049
T_PREF_left_FD	0.047
T_PREF_right_FD	0.037
T_PREM_left_FD	0.027
CST_left_fdc	0.050
FPT_left_fdc	0.015
FPT_right_fdc	0.013
T_PREM_left_fdc	0.049

*Note*: Age, sex, education, SDS score, and SAS score are included as covariates. (*P* < 0.05).

Abbreviations: ATR, Anterior thalamic radiation; CST, Corticospinal tract; FD, Fiber density; FDC, Fiber density and cross‐section; FPT, Fronto‐pontine tract; MLF, Middle longitudinal fascicle; POPT, Parieto‐occipital pontine; SCP, Superior cerebellar peduncle; ST_PREF, Striato‐prefrontal; T_PAR, Thalamo‐parietal; T_POSTC, Thalamo‐postcentral; T_PREC, Thalamo‐precentral; T_PREF, Thalamo‐prefrontal; T_PREM, Thalamo‐premotor.

**TABLE 3:** Differences among the PTSD group, the TEC group, and the HC group are shown in fiber tracts.

	Fiber tract	Adjust value of *P* (Bonferroni)
**FD**		
PTSD vs. HC	FPT_right	0.003**
	MLF_right	0.002**
TEC vs. HC	None	
PTSD vs. TEC	None	
**FC**		
PTSD vs. HC	None	
TEC vs. HC	None	
PTSD vs. TEC	None	
**FDC**		
PTSD vs. HC	FPT_left	<0.001**
	FPT_right	<0.001**
	T_PREM_left	0.002**
TEC vs. HC	None	
PTSD vs. TEC	None	

Abbreviations: FC, the cross‐section of fiber bundles; FD, fiber density; FDC, fiber density and cross‐section; FPT, fronto‐pontine tract; HC, health control; MLF, middle longitudinal fascicle; PST, post sumo trit; PTSD, posttraumatic stress disorder; TEC, controls exposed to trauma; T_PREM, thalamo‐premotor.

**p < 0.01 showed marked distinctions.

### 3.4 Classification in Machine Learning

No statistically significant differences were detected between the PTSD and TEC groups using the above statistical approaches. Because distinguishing the two or three groups remains challenging, machine learning methods were applied to further explore group differences. Binary and multiclass classification models were established to discriminate PTSD from TEC and to differentiate PTSD and TEC from HC. During feature selection using the recursive feature elimination with cross‐validation (RFECV) approach, features were ranked according to their importance, and the least informative feature was removed at each iteration (stepwise = 1). This process continued until all features had been evaluated. To determine the optimal number of features with the highest predictive performance, models with different feature counts were cross‐validated using recursive feature elimination (RFE). The feature selection process was then finalized based on the model with the best average performance.

**FIGURE 3:**
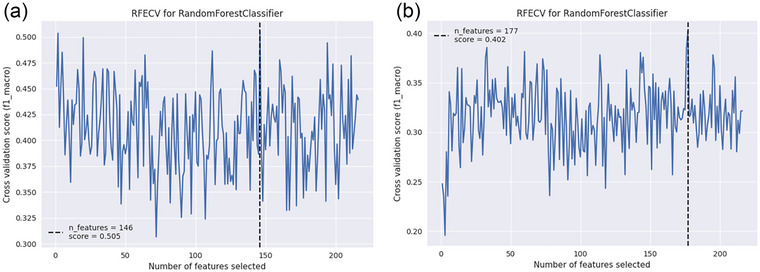
Feature selection performance of the Random Forest Classifier using Recursive Feature Elimination with Cross‐Validation (RFECV) across PTSD, TEC, and HC groups. (a) shows the performance of RFECV for the Random Forest Classifier in selecting features that are important for PTSD (PTSD compared to TEC). All 216 features were calculated with the use of diverse estimators. The score of 0 was the highest one achieved by the Random Forest Classifier. 505 by choosing 146 properties An analysis of the performance when Recursive Feature Elimination with Cross‐Validation (RFECV) is used for Random Forest Classifiers to pick out significant features related to PTSD. (PTSD vs. TEC vs. HC). The Random Forest Classifier, having 177 features selected, achieved a better performance compared to other classification methods, with a f1 score of 0.402.

**FIGURE 4:**
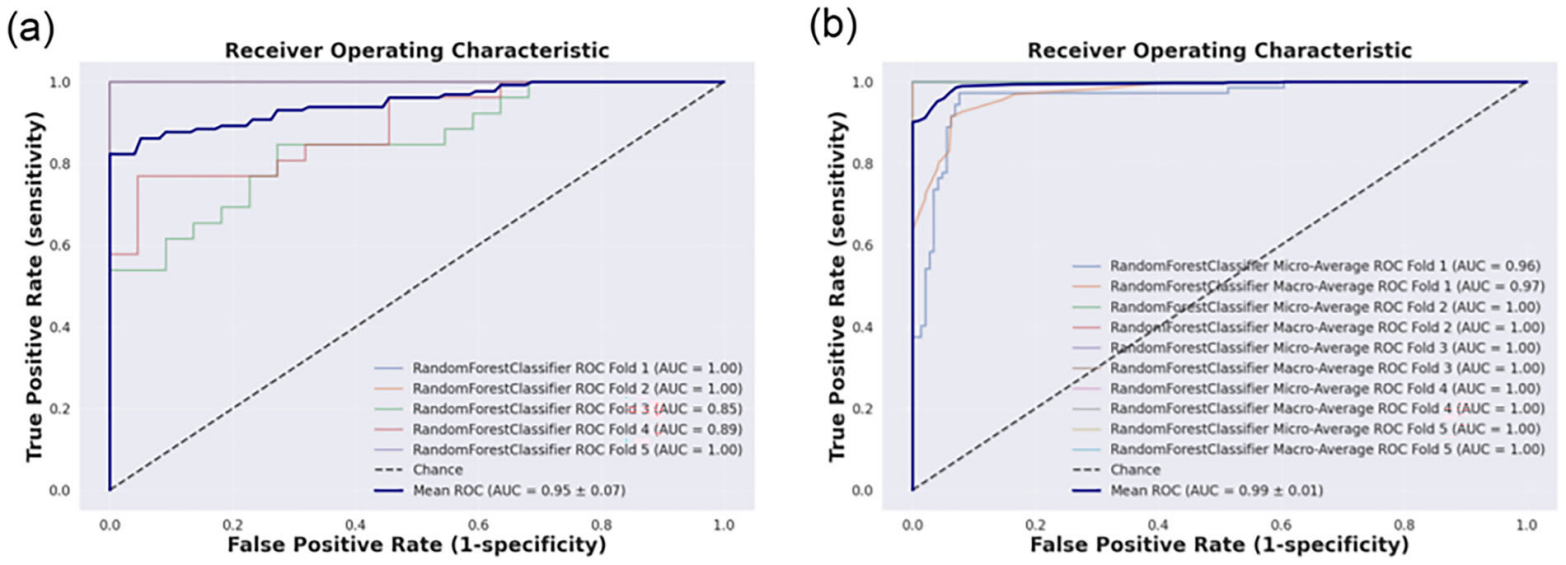
Receiver Operating Characteristic (ROC) curves of the machine learning models for PTSD classification (PTSD vs. TEC vs. HC) using five‐fold cross‐validation. (a) presents the ROC curves for the machine learning model (PTSD vs. the 5 ‐ fold cross‐validation results of TEC are presented; (b) the ROC curves for the machine‐learning model in the case of PTSD are presented. TEC vs. Shown are the ROC curves for the machine‐learning model (PTSD vs. HC) with 5‐fold cross‐validation. For every fold model in multiple classification models, the macro‐average Receiver Operating Characteristic (ROC) and the micro‐average ROC are calculated, respectively.

**FIGURE 5:**
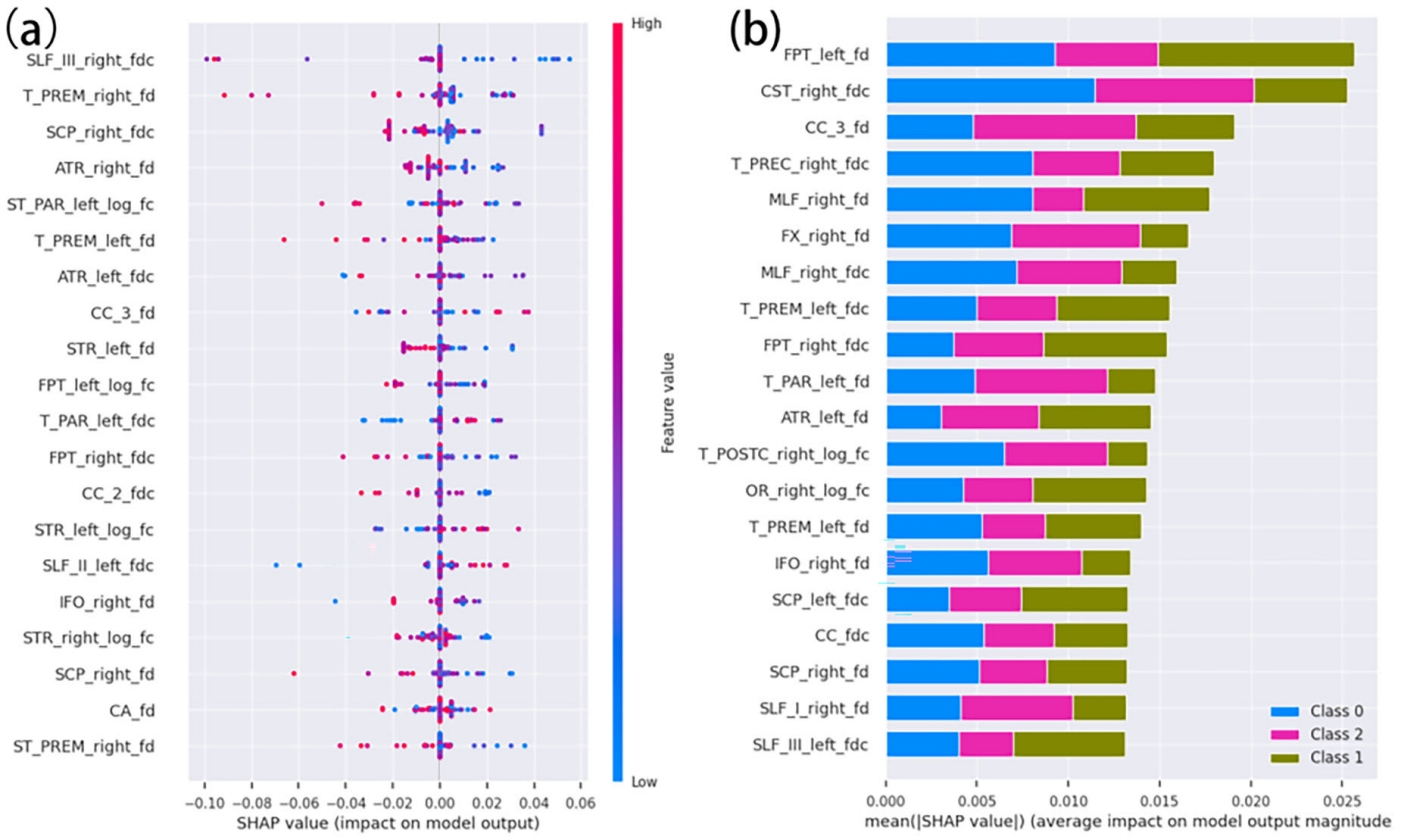
Top 20 important feature predictors ranked by mean absolute SHAP values in machine learning models for PTSD classification (PTSD vs. TEC and multiclass models). (a) shows the Absolute Shapley Additive Explanations (SHAP). Values of the top 20 crucial feature predictors in the machine learning model (PTSD vs. TEC); (b) Among the multi‐classification machine learning models, the top 20 key feature predictors are those with the largest mean of absolute SHAP values. Namely, the sum of the absolute SHAP value in each classification is the highest. The third part of the superior longitudinal fascicle, which is named SLF III; The T_PREM refers to Thalamo‐premotor. The superior cerebellar peduncle is abbreviated as SCP. ATR stands for Anterior thalamic radiation. ST_PAR refers to Striato‐parietal. CC 3 Rostral body: The superior thalamic radiation is denoted as STR. The fronto‐pontine tract, abbreviated as FPT. CC 2, Genu; SLF II stands for Superior longitudinal fascicle II; the Inferior Occipito‐Frontal Fascicle, abbreviated as IFO. The anterior commissure, abbreviated as CA. The Striato‐premotor can be abbreviated as ST_PREM.The Corticospinal tract, abbreviated as CST, T_PREC stands for Thalamo‐Precentral; The Middle longitudinal fascicle is abbreviated as MLF. FX, Fornix; T_PAR, Talamo‐parietal; T_POSTC represents Thalamo‐postcentral. or optical radiation; the Inferior cerebellar peduncle is denoted as ICP; The Rostrum of the Corpus Callosum, denoted as CC; I is for SLF, which stands for Superior Longitudinal Fascicle.

For PTSD vs. TEC, the optimal 146‐dimensional feature set was identified using recursive feature elimination (RFE, stepwise = 1) based on the Macro‐F1 score. The classification performance did not improve substantially with an increasing number of features (Figure 3a). Based on these selected features, a Random Forest (RF) classifier with five‐fold cross‐validation achieved an accuracy of 0.89, sensitivity of 0.97, specificity of 0.71, precision of 0.87, and an area under the curve (AUC) of 0.95 (Figure 4a). For PTSD vs. TEC vs. HC, the RFE approach based on the Macro‐F1 score yielded the optimal classifier when 177 features were selected (Figure 3b). The three‐class model demonstrated excellent discriminative performance, achieving a macro‐averaged precision, recall, and F1‐score of 0.99, indicating balanced classification across diagnostic categories. Weighted‐ and micro‐averaged metrics were similarly high, suggesting robust generalization without class‐size bias (Table S1). A Random Forest classifier with five‐fold cross‐validation further confirmed the model's stability, with mean precision and recall of 0.99 and one‐vs‐rest AUC values ranging from 0.96 to 1.00 across folds (Figure 4b). The top 20 features contributing most to classification performance in the binary model are shown in Figure 5a, representing those with the highest SHAP value proportions. Figure 5b displays the top 20 features of the multiclass model with the greatest mean absolute SHAP values, corresponding to the highest summed SHAP contributions within each classification category.

## 4 Discussion

This study investigated whether individuals with typhoon‐related PTSD exhibit common abnormalities in white matter (WM) structural networks. A whole‐brain fixel‐based analysis (FBA) identified specific WM tracts showing group‐level differences in fiber density (FD) and fiber cross‐section (FC) among the PTSD, TEC, and HC groups. Significant FD differences were observed in the right frontopontine tract (FPT) and right middle longitudinal fasciculus (MLF), and FDC alterations were found in the bilateral FPT and left thalamo‐premotor tract (T_PREM) in the PTSD group compared with healthy controls. A machine learning classification model further demonstrated high accuracy in distinguishing PTSD from TEC and HC, suggesting the potential diagnostic utility of FBA‐derived features.

This study examined white matter alterations associated with typhoon‐related PTSD from a network‐level perspective. PTSD is increasingly conceptualized as a disorder of distributed brain networks rather than focal regional pathology, involving disrupted integration across prefrontal, subcortical, and associative systems that support emotion regulation, attentional control, and sensorimotor processing. Within this framework, white matter alterations are expected to be subtle, spatially distributed, and anatomically coherent across long‐range fiber pathways rather than confined to isolated tracts or regions.

In the whole‐brain fixel‐wise analysis, no group differences survived family‐wise error correction. This finding is not unexpected given the extremely large number of fixels assessed and the conservative nature of fixel‐level FWE correction. Importantly, the spatial distribution of uncorrected effects demonstrated a coherent pattern across the FPT, MLF, and T_PREM (Figure S1–6), which closely aligned with subsequent tract‐based ROI findings (Table 2). This pattern suggests that the observed alterations are spatially consistent but of modest effect size, a characteristic commonly reported in psychiatric disorders with network‐level pathology. Accordingly, the tract‐based ROI analysis was employed as an anatomically informed aggregation strategy to enhance sensitivity while preserving fiber‐specific information.

Compared with our previous study based on automated fiber quantification (AFQ) (Zhang et al. 2023), the current findings—particularly in the right anterior thalamic radiation (ATR)—show good consistency with earlier results obtained from the same cohort. However, the outcomes from AFQ and FBA are not identical, largely due to methodological differences. AFQ adopts deterministic tractography, whereas FBA employs probabilistic modeling, which captures complex crossing fibers that deterministic tracking may overlook. Despite these technical differences, both methods revealed partially overlapping alterations, indicating the robustness of the findings. In contrast, automated fiber quantification (AFQ) primarily relies on diffusion tensor–derived scalar metrics, such as FA and MD, or tract‐averaged profiles, which are susceptible to signal mixing in regions of complex fiber architecture. By disentangling micro‐ and macrostructural changes at the sub‐bundle level, FDA provides greater sensitivity and mechanistic insight, making it particularly well suited for investigating non‐focal, network‐level white matter alterations characteristic of PTSD.

These findings extend previous results from the same cohort, in which diffusion indices such as fractional anisotropy (FA), axial diffusivity (AD), and radial diffusivity (RD) were analyzed using voxel‐based analysis (VBA) (Chen et al. 2021) and automated fiber quantification (AFQ) based on deterministic tractography (Zhang et al. 2023). The present results partially align with earlier studies and support the hypothesis that altered thalamo‐cortical connectivity contributes to the neuropathophysiology of PTSD. The ATR, connecting the thalamus and prefrontal cortex, plays an essential role in emotional regulation (Spalletta et al. 2013). Although not classically considered part of the limbic emotional circuit, its involvement in PTSD may contribute to impaired memory retention and persistent hyperarousal or fear responses (Yin et al. 2011). Recent evidence further suggests that the prefrontal cortex, rather than the hippocampus, stores long‐term memories of recent fears (Lee et al. 2023). The involvement of the FPT and T_PREM—tracts linking the prefrontal cortex with subcortical structures—supports the view that the prefrontal cortex is central to long‐term PTSD memory consolidation. These findings enhance our understanding of the spatial distribution of WM microstructural abnormalities in PTSD and highlight potential neuroimaging biomarkers for its diagnosis.

### 4.1 Biological and Transdiagnostic Interpretation of White‐Matter Alterations

While the present findings are derived from advanced diffusion imaging and network‐level analyses, their interpretation benefits from integration with emerging biological evidence from trauma research. A growing body of literature demonstrates that traumatic stress is associated with systemic oxidative imbalance, oxidative DNA damage, and disrupted redox regulation, as reported not only in PTSD (Kurhan and Alp 2021b) but also in other stress‐related psychiatric conditions such as obsessive–compulsive disorder (Kurhan et al. 2022). Comparable alterations in oxidative stress markers have also been observed in attention‐deficit/hyperactivity disorder, further supporting the notion of shared stress‐related biological vulnerability across diagnostic categories (Kurhan and Alp 2021a; Kurhan et al. 2022). Such biological dysregulation may contribute to axonal vulnerability, neuroinflammatory processes, and altered long‐range connectivity, thereby providing a mechanistic backdrop for the observed increases in fiber density and fiber density–cross section within prefrontal–subcortical and thalamo‐cortical pathways.

Importantly, similar structural and microstructural alterations have been reported across multiple psychiatric conditions. Recent studies documenting retinal and neuroanatomical changes in psychosis and bipolar disorder highlight that structural deviations are often transdiagnostic in nature, reflecting shared vulnerability pathways rather than disorder‐specific markers (Kurhan et al. 2026; Seven and Kurhan 2025). Within this framework, the white‐matter alterations identified in the present study should be interpreted as dimensional neurobiological signatures associated with stress exposure and vulnerability, rather than as exclusive features of PTSD.

This transdiagnostic perspective is particularly relevant for interpreting the machine‐learning results. The high classification performance observed in this study likely reflects the model's sensitivity to distributed stress‐related neurobiological patterns embedded in white‐matter networks. Such patterns may index vulnerability or resilience following trauma exposure, rather than providing definitive disorder‐specific biomarkers. Accordingly, our findings align with contemporary dimensional models of psychopathology and emphasize the importance of cautious interpretation when applying machine‐learning techniques to modestly sized clinical samples.

### 4.2 Tract‐Level Interpretation of White Matter Alterations

#### 4.2.1 Frontopontine Tract (FPT)

In this study, after Bonferroni correction, the FPT showed significant FD and FDC increases in PTSD compared with healthy controls. The FPT originates in the frontal cortex and terminates in the pontine nuclei, forming part of the corticopontine system (Leergaard and Bjaalie 2007). It descends through the anterior limb of the internal capsule, linking the cortex with the cerebellum and contributing to motor coordination. Its involvement in lexico‐semantic aspects of action naming has been reported in aphasic patients (Akinina et al. 2019). These findings are consistent with previous observations of internal capsule and corona radiata alterations in the same cohort (Chen et al. 2021).

#### 4.2.2 Middle Longitudinal Fasciculus (MLF)

PTSD patients also showed increased FA in the right MLF. This tract connects the superior temporal gyrus (STG) with the angular gyrus (AG) and superior parietal lobule (SPL) (Makris et al. 2017). The STG–AG connection supports language and attentional functions, whereas the STG–SPL connection contributes to audiovisual and visuospatial integration (Makris et al. 2013). Structural abnormalities in these pathways have been linked to impaired semantic processing, with negative correlations observed between WM integrity and word comprehension and naming performance (Luo et al. 2020).

#### 4.2.3 Thalamo‐Premotor Tract (T_PREM)

The left T_PREM showed significantly elevated FDC in PTSD. As a key component of the cerebello‐thalamo‐cortical (CTC) system, this tract supports voluntary motor control (Nashef et al. 2018; Nashef et al. 2018). Abnormalities in T_PREM may increase sensitivity to external stimuli, especially when motor symptoms are prominent (Von Podewils et al. 2015). Catatonic patients have shown increased FA in this tract (Sasada et al. 2017), supporting a link between T_PREM dysconnectivity and abnormal sensorimotor responsiveness.

#### 4.2.4 Superior Longitudinal Fasciculus III (SLF‐III)

Machine‐learning classification revealed the right SLF‐III as the strongest discriminator between PTSD and TEC. SLF‐III (the ventral SLF) connects the inferior parietal lobule with the inferior frontal gyrus, ventral premotor cortex, and precentral gyrus (Nakajima et al. 2020). It contributes to self‐face recognition and proprioceptive illusion processing (Morita et al. 2017) and plays a central role in stimulus‐driven (exogenous) attentional control. Its involvement aligns with clinically observed hypervigilance in PTSD (Klarborg et al. 2013; Martín‐Signes et al. 2019).

#### 4.2.5 Superior Cerebellar Peduncle (SCP)

Increased FD in the right SCP highlights the cerebellum's role in PTSD. Although traditionally overlooked in fear neurocircuitry, trauma‐exposed individuals show altered cerebellar connectivity with emotion‐regulatory networks (Blithikioti et al. 2022). SCPs form the major cerebellar efferent pathway (Hanaie et al. 2013; Rasmussen 1933), projecting from the dentate nucleus to the red nucleus, thalamus, reticular formation, and other subcortical structures (Hans 2011; Kitamura et al. 2008; Salamon et al. 2007; Wakana et al. 2004). Reduced SCP integrity has been associated with impaired proprioceptive weighting and postural control (Manto et al. 2012), suggesting altered sensorimotor integration in PTSD.

#### 4.2.6 Thalamo‐Parietal and Other Thalamic Tracts (T_PAR)

Both T_PREM and T_PAR displayed increased FBA (Wakana et al. 2004) values. Thalamic volume and tract integrity are associated with motor and verbal memory performance, and structure–function correlations have been reported between T_PREM and motor execution (Philp et al. 2014). WM tracts involving the thalamus, cingulum, and corpus callosum contribute to motor, cognitive, and emotional regulation (Monti et al. 2015; Phillips et al. 2003; Schirinzi et al. 2016). Thalamic pathways linking the frontal cortex and limbic regions mediate motor–emotional coupling, influencing facial expression and mood regulation (Niu et al. 2020). Cognitive control deficits related to these thalamo‐cortical circuits are frequently observed in psychiatric disorders (Miao et al. 2019).

However, significant PTSD–HC differences were identified in several tracts; their FBA metrics were not significantly correlated with symptom severity. This likely reflects the limited symptom range and modest sample size and does not preclude their clinical relevance. White matter alterations may indicate broader network‐level features or vulnerability markers rather than direct expressions of symptom intensity.

To sum up, the machine‐learning results help clarify why some trauma‐exposed individuals develop PTSD whereas others remain resilient. The SLF‐III, SCP, and prefronto‐subcortical pathways (FPT, T_PREM) emerged as the most discriminative features, reflecting networks involved in stimulus‐driven attention, emotional regulation, and top‐down control. Alterations in these circuits were observed in PTSD but not in TEC, suggesting that vulnerability may arise from heightened bottom‐up reactivity and reduced regulatory integration, whereas resilience may depend on the preservation of these pathways.

## 5 Limitations

There are several limitations to this study. First, it employed a cross‐sectional design with a relatively small sample size. Although sufficient for this initial exploratory study, the limited sample size may reduce statistical power and generalizability of the findings. Future research should include larger cohorts to validate our results. Second, the limited sample size may have increased the likelihood of false‐negative findings; however, machine‐learning techniques were applied to enhance sensitivity to subtle and distributed group differences. We acknowledge that a larger sample would provide more robust and reliable estimates of the effect sizes. Third, our diagnoses were based on the DSM‐IV criteria, as the study was initiated prior to the publication of DSM‐5. However, the core symptomatology of re‐experiencing, avoidance, and hyperarousal remains central to the diagnosis across both systems. Future studies should employ the most up‐to‐date diagnostic criteria to ensure contemporary clinical relevance and facilitate direct comparison with ongoing research. Fourth, the generalizability of our findings may be limited by the specific trauma type studied. Our cohort consisted exclusively of survivors of a single natural disaster (a typhoon). It remains to be determined whether the identified FBA features or imaging patterns extend to PTSD resulting from other traumatic experiences, such as interpersonal violence or combat. Future research involving diverse trauma‐exposed populations is needed to ascertain the broader applicability of our results. Fifth, current FBA analyses rely on the MRtrix3 platform, which applies family‐wise error (FWE) correction at the fixel level. The large number of statistical comparisons required may have contributed to the absence of whole‐brain FWE‐corrected results. Despite these methodological constraints, the expanding understanding of white matter involvement in neuropsychiatric disorders highlights the need for continued refinement of diffusion‐based analytical approaches. Finally, although years of education were included as a covariate, the significant difference in education level between the healthy control group and the clinical groups (PTSD and TEC) remains a potential confounding factor. Future studies should include groups matched on socioeconomic and educational backgrounds to better validate the specificity of the present findings. In addition, the neurobiological alterations identified in this study should be interpreted in the context of emerging transdiagnostic perspectives in psychiatry. Structural and microstructural changes in white matter have been observed across multiple psychiatric conditions, indicating that some diffusion‐based features may capture broadly shared stress‐related or vulnerability‐related neural processes. Importantly, this does not detract from the relevance of the present findings for PTSD but highlights the value of future larger and longitudinal studies in further characterizing the specificity and overlap of trauma‐related neurobiological patterns and in strengthening the interpretation of machine‐learning–based neuroimaging models.

## 6 Conclusions

Individuals with typhoon‐related PTSD exhibited distinct white matter alterations compared with TEC and HC. By integrating fixel‐based analysis with machine‐learning techniques, this study identified PTSD, TEC, and HC groups. The three‐class classification model further demonstrated that diffusion‐derived white matter features capture meaningful neurobiological differences among PTSD, TEC, and HC groups. Together, these findings advance understanding of the network‐level white matter alterations associated with PTSD and provide a proof‐of‐concept framework for future studies investigating imaging markers of vulnerability and resilience following traumatic exposure.

## Author Contributions

Y. Z., H. C. and F. C. contributed to the conception and design of this study. Y. Z., H. C., R. Q., J. K., and F. C performed the statistical analysis and wrote the manuscript. Y. Z., H. C., R. Q., J. K., Q. X., Y. Z., L. Z. and G. L. performed the experiments. Y. Z. wrote the first draft of the manuscript.

## Conflicts of Interest

The authors declare no conflicts of interest.

## Supporting information




**Supplementary**: brb371354‐sup‐0001‐SuppMat.docx

## Data Availability

The datasets used during the current study are available from the corresponding author on reasonable request.
