# Supplementary material for: A Diagnosis Model of Typhoon‐Related Post‐Traumatic Stress Disorder Based on Fixel‐Based Analysis in Machine Learning
Source: Brain Behav. 2026 Apr 6;16(4):e71354. doi: 10.1002/brb3.71354 (PMC13053301; doi:10.1002/brb3.71354)
Supplement: Supplementary file 1 — Supplementary: brb371354‐sup‐0001‐SuppMat.docx [file BRB3-16-e71354-s001.docx]

Supplementary


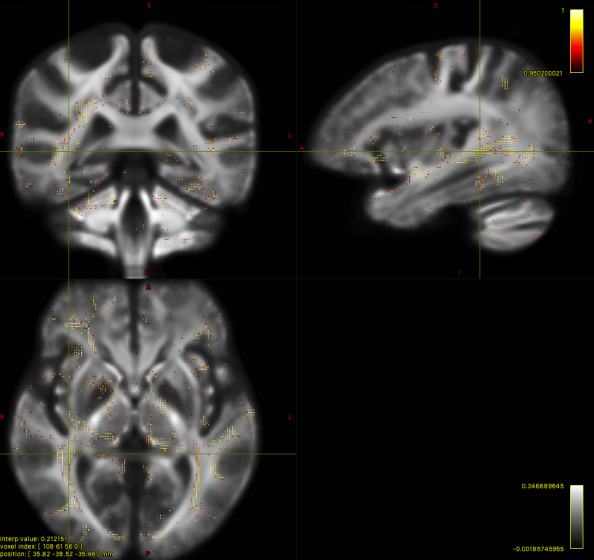

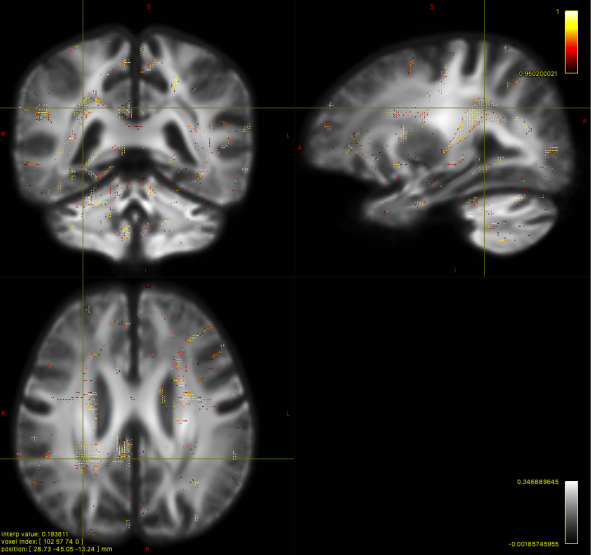


Supplementary Fig.1-2 FD statistical results


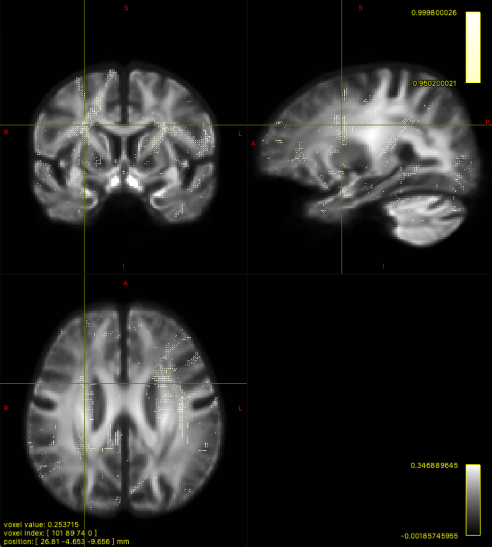

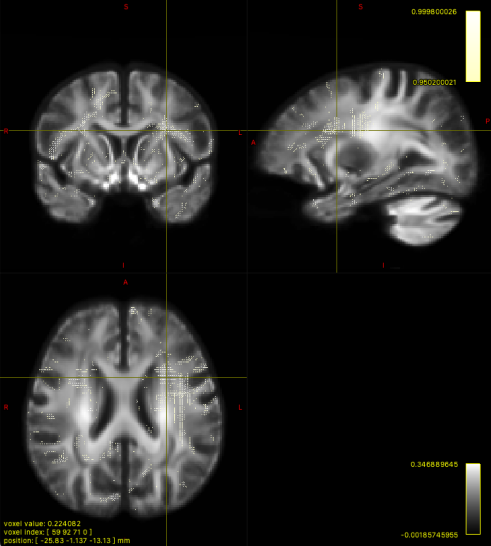


Supplementary Fig.3-4 FDC statistical results


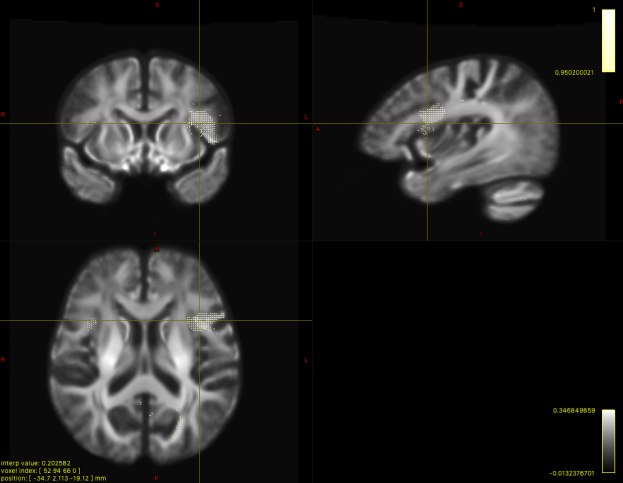

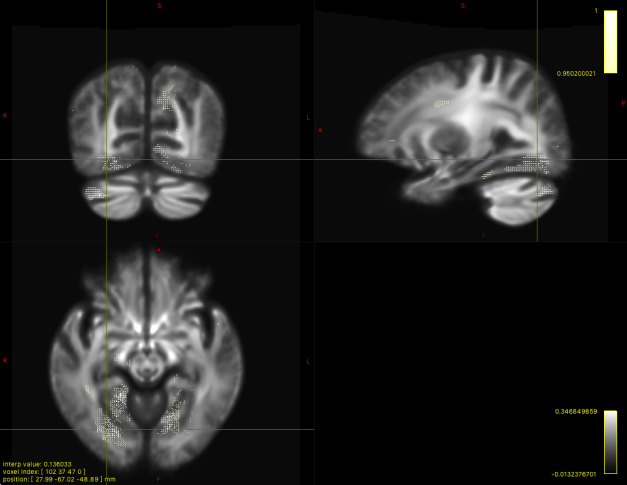


Supplementary Fig.5-6 FC statistical results

Table S1. Classification performance metrics of the three-class model

|  | **f1-score** | **precision** | **recall** |
| --- | --- | --- | --- |
| macro avg | 0.994 | 0.995 | 0.994 |
| weighted avg | 0.994 | 0.995 | 0.994 |
| micro avg | 0.994 | 0.994 | 0.994 |
